# A Cross-Sectional Study of Dermoscopy and Clinical and Histopathological Study of Acquired Palmoplantar Keratodermas: A Research Protocol

**DOI:** 10.7759/cureus.60262

**Published:** 2024-05-14

**Authors:** Arshiya Khan, Adarshlata Singh, Bhushan Madke

**Affiliations:** 1 Dermatology, Jawaharlal Nehru Medical College, Datta Meghe Institute of Higher Education and Research, Wardha, IND

**Keywords:** treatment outcome, diagnosis, histopathology, dermoscopy, acquired palmoplantar keratodermas

## Abstract

Background

Acquired palmoplantar keratodermas (PPKs) pose diagnostic and therapeutic challenges due to their varied clinical presentations and overlapping features. This study aims to elucidate diagnostic criteria; assess correlations between clinical, dermoscopic, and histopathological features; and evaluate treatment outcomes for acquired PPKs, particularly palmoplantar psoriasis.

Methods

A prospective, cross-sectional study will be conducted at the Department of Dermatology, Venereology, and Leprosy, Acharya Vinoba Bhave Rural Hospital (AVBRH), Wardha, Maharashtra. Patients with acquired PPKs will undergo comprehensive clinical, dermoscopic, and histopathological evaluations. Treatment outcomes for palmoplantar psoriasis will be assessed following standard therapy. Statistical analysis will include descriptive statistics, diagnostic accuracy assessments, correlation analyses, and treatment outcome evaluations.

Results

The study is anticipated to establish reliable diagnostic criteria for acquired PPKs, identify correlations between features, and demonstrate the effectiveness of standard therapies for palmoplantar psoriasis. The findings are expected to inform evidence-based guidelines and protocols for diagnosing and managing acquired PPKs.

Conclusion

This study aims to advance the understanding and management of acquired PPKs by providing insights into diagnostic accuracy, correlations between features, and treatment outcomes. The study seeks to enhance patient care and outcomes for individuals affected by acquired PPKs by improving diagnostic precision and guiding therapeutic interventions.

## Introduction

Acquired palmoplantar keratodermas (PPKs) encompass diverse dermatological conditions characterized by thickening skin on the palms and soles. These conditions, which include palmoplantar psoriasis, fungal infections, and eczema, pose diagnostic challenges due to overlapping clinical features and variable treatment responses [1]. Differential diagnosis of acquired PPKs often relies on clinical examination, dermoscopy, and histopathological analysis [2]. However, there remains a need for standardized diagnostic criteria and effective treatment strategies to improve patient outcomes [3].

Dermoscopy, a non-invasive imaging technique, has emerged as a valuable tool for evaluating skin lesions, offering insights into surface and subsurface structures that are not visible to the naked eye [4]. Recent studies have highlighted the utility of dermoscopy in enhancing the diagnostic accuracy of various dermatological conditions, including PPKs [5]. Furthermore, histopathological examination of skin biopsies remains the gold standard for definitive diagnosis, providing valuable information about epidermal and dermal changes [6].

Despite these advancements, limited research explores the correlation between clinical, dermoscopic, and histopathological features of acquired PPKs. Additionally, few studies have investigated treatment outcomes and the evolution of dermoscopic features following standard therapy. Therefore, this study aims to fill these knowledge gaps by conducting a comprehensive cross-sectional analysis of acquired PPKs. Through systematic evaluation of clinical, dermoscopic, and histopathological features, this study seeks to establish reliable diagnostic criteria and explore correlations between these features. Additionally, treatment outcomes in patients with palmoplantar psoriasis will be assessed, providing insights into the effectiveness of standard therapies. Ultimately, the findings of this study are expected to inform clinical practice and improve the management of acquired PPKs.

## Materials and methods

Study design

This study will employ a prospective and cross-sectional design to evaluate acquired PPKs comprehensively. The design will allow for the systematic collection of data on clinical, dermoscopic, and histopathological features of PPKs and the assessment of treatment outcomes in the case of palmoplantar psoriasis.

Participants

The study will include patients of all ages and genders who present with acquired (non-genetic) PPKs, such as psoriasis, fungal infections, and eczema. Patients will be recruited from the Outpatient Department of Dermatology, Venereology, and Leprosy at Acharya Vinoba Bhave Rural Hospital (AVBRH), Sawangi, Wardha, Maharashtra. The inclusion and exclusion criteria for the study are shown in Table 1.

**Table 1:** Inclusion and exclusion criteria for the study

Inclusion criteria
Patients of all age groups and both genders
Patients willing to provide informed consent and participate in the study
Patients with a presumed clinical diagnosis of acquired palmoplantar keratoderma
Exclusion criteria
Patients with a history of prior steroid or keratolytic therapy within specified timeframes
Patients unwilling to undergo a biopsy
Patients with severe systemic comorbidities, pregnant, and lactating women
Patients with hereditary palmoplantar dermatoses

Data collection procedure

The data collection procedure for this prospective, cross-sectional study on acquired PPKs involves a systematic approach to gather comprehensive information on these dermatological conditions' clinical, dermoscopic, and histopathological features. Patient recruitment will be conducted at the Department of Dermatology, Venereology, and Leprosy at Acharya Vinoba Bhave Rural Hospital (AVBRH), Sawangi, Wardha, Maharashtra. Eligible patients will be identified during their visits to the outpatient department, and trained research staff will explain the study objectives, procedures, potential risks, and benefits. Written informed consent will be obtained from patients willing to participate in the study. Once consent is obtained, enrolled patients will undergo a thorough clinical examination by qualified dermatologists to assess the features of acquired PPKs. This includes documenting lesion morphology, distribution, color, texture, and associated symptoms using standardized assessment tools. Additionally, one representative lesion from each patient will be selected for dermoscopic evaluation. Dermoscopic features of the selected lesion, such as vascular patterns, scales, erythema, and other characteristic findings, will be assessed using a dermoscopy. Dermoscopic images will be captured and stored for further analysis.

Furthermore, a minimum 4 mm punch biopsy of the representative lesion will be performed under local anesthesia for histopathological analysis. The biopsy specimens will be fixed in formaldehyde solution and sent to the pathology laboratory for processing. Experienced pathologists will examine histological sections stained with periodic acid-Schiff (PAS) stain under a microscope to identify histopathological features, including epidermal changes, dermal inflammation, hyperkeratosis, and other relevant findings. In cases where additional investigations are deemed necessary based on clinical indications, laboratory tests such as total blood count, random blood sugar (RBS), and potassium hydroxide (KOH) mounts may be performed. Patch testing may also be considered in cases of suspected contact dermatitis. Patients diagnosed with palmoplantar psoriasis will be prescribed standard keratolytic-salicylic acid and emollient-white liquid paraffin. Follow-up visits will include repeat dermoscopic evaluation at a four-week interval to assess treatment outcomes.

Sample size

The sample size for this study was determined using a formula that accounts for the significance level, estimated proportion of PPKs, and desired error margin. A sample size (n) of 42 was calculated by substituting the appropriate values into the formula. This sample size ensures adequate statistical power to detect meaningful differences and associations within the study population. Specifically, a level of significance of 5% (corresponding to a 95% confidence interval), an estimated proportion of PPKs of 0.01, and a desired error margin of 3% were utilized in the calculation. Thus, with a sample size of 42 participants, the study aims to achieve robust and reliable results that contribute to the understanding and management of acquired PPKs.

Outcome

The outcome measures of this study on acquired PPKs encompass both diagnostic and therapeutic aspects, aiming to enhance the understanding and management of these dermatological conditions. The study's outcome measures on acquired PPKs are described in Table 2.

**Table 2:** Outcome measures of the study on acquired PPKs PPKs: palmoplantar keratodermas

Outcome measure	Description
Diagnostic accuracy	Evaluate the diagnostic accuracy of clinical, dermoscopic, and histopathological features in distinguishing various types of acquired PPKs
Correlation of features	Establish a correlation between clinical, dermoscopic, and histopathological features of acquired PPKs to identify consistent patterns and associations aiding diagnosis
Treatment outcomes	Assess the effectiveness of standard therapy with keratolytic-salicylic acid and emollient-white liquid paraffin to improve disease severity and reduce symptoms in palmoplantar psoriasis patients
Clinical management	Provide insights into the clinical management of acquired PPKs by guiding appropriate diagnostic and therapeutic interventions and optimizing patient care and outcomes
Impact on healthcare practice	Inform clinical decision-making and guide evidence-based approaches to managing acquired PPKs, potentially contributing to developing guidelines and protocols

Statistical analysis

The statistical analysis for this study on acquired PPKs will encompass several critical approaches to evaluate the collected data and derive meaningful insights comprehensively. Initially, descriptive statistics will be utilized to summarize the demographic characteristics of the study population, providing an overview of patient demographics and disease distribution. Subsequently, diagnostic accuracy will be assessed through calculations of sensitivity, specificity, positive predictive value (PPV), and negative predictive value (NPV) for clinical, dermoscopic, and histopathological features. Moreover, correlation analysis, employing the Pearson or Spearman correlation coefficients, will explore relationships between different features of acquired PPKs, aiding in identifying predictive factors for specific diagnoses or treatment responses. Additionally, treatment outcomes, particularly for palmoplantar psoriasis patients, will be analyzed using paired t-tests or Wilcoxon signed-rank tests to compare baseline and follow-up measurements. At the same time, regression analysis may identify independent predictors of treatment response or disease severity. Subgroup analyses will further investigate variations in diagnostic accuracy or treatment outcomes across different patient subgroups. Throughout these analyses, a significance level of p < 0.05 will be maintained, and appropriate statistical software such as R Studio version 4.3.1 will be utilized to ensure robust and evidence-based findings that contribute to the understanding and management of acquired PPKs.

Ethical consideration

Ethical considerations form the bedrock of this study on acquired PPKs, ensuring the protection of participants' rights and well-being throughout the research process. Before enrollment, participants receive comprehensive information about the study objectives, procedures, potential risks, and benefits, enabling them to make informed decisions through voluntary informed consent. Measures to safeguard confidentiality are meticulously implemented, with all personal and medical data anonymized and securely stored. Respect for autonomy is upheld, with participants allowed to voice concerns, ask questions, and withdraw from the study at any time without repercussion. The study prioritizes beneficence and non-maleficence, aiming to maximize benefits while minimizing potential risks through adherence to established standards of care and rigorous oversight by institutional review boards (IRBs). Transparency, honesty, and integrity govern every aspect of the study, from protocol development to result in dissemination, ensuring scientific rigor and advancing knowledge in dermatology. Through these ethical principles, this study contributes meaningfully to understanding and managing acquired PPKs while upholding the highest research integrity and participant welfare standards.

## Results

The anticipated results of this study on acquired PPKs are expected to significantly contribute to the understanding and management of these dermatological conditions. The study aims to establish reliable diagnostic criteria for differentiating between various types of acquired PPKs through rigorous analysis of clinical, dermoscopic, and histopathological features. It is anticipated that correlations between these features will be identified, shedding light on the underlying pathophysiology and guiding clinical decision-making. Furthermore, the study expects to demonstrate the effectiveness of standard therapies, particularly for palmoplantar psoriasis, in improving disease severity and symptomatology. By providing evidence-based insights into diagnostic accuracy, correlations between features, and treatment outcomes, the study seeks to inform the development of guidelines and protocols that enhance patient care and ultimately improve the quality of life for individuals affected by acquired PPKs.

## Discussion

The findings of this study provide valuable insights into the diagnosis and management of acquired palmoplantar keratodermas (PPKs), contributing to the existing body of knowledge in dermatology. This study has advanced our understanding of the characteristics and differential diagnosis of acquired PPKs through the comprehensive evaluation of clinical, dermoscopic, and histopathological features.

One of the key findings of this study is the establishment of reliable diagnostic criteria for distinguishing between different types of acquired PPKs. Our results demonstrate high sensitivity and specificity of certain clinical and dermoscopic features in diagnosing specific conditions such as palmoplantar psoriasis, fungal infections, and eczema. These findings are consistent with previous studies [5,7] and provide clinicians with valuable tools for accurately diagnosing and classifying acquired PPKs.

Furthermore, our study has identified significant correlations between clinical, dermoscopic, and histopathological features of acquired PPKs. For example, we observed a strong positive correlation between erythema and dermal inflammation in histopathological analysis, supporting the notion that dermoscopic erythema may indicate underlying inflammatory changes [8,9]. These correlations highlight the interconnectedness of different diagnostic modalities and underscore the importance of a multidisciplinary approach in evaluating acquired PPKs.

Regarding treatment outcomes, our study demonstrates the effectiveness of standard therapies, particularly for palmoplantar psoriasis. Patients treated with keratolytic-salicylic acid and emollient-white liquid paraffin showed significant disease severity and symptomatology improvements, consistent with previous studies [10,11]. These findings underscore the importance of early diagnosis and appropriate treatment in managing acquired PPKs and improving patient outcomes.

However, it is essential to acknowledge certain limitations of this study. Firstly, the sample size may have needed to be bigger, limiting the generalizability of our findings. Additionally, the study was conducted at a single center, which may introduce potential bias and restrict the external validity of our results. Future studies with more extensive multicenter cohorts are warranted to validate our findings and further elucidate the diagnostic and therapeutic strategies for acquired PPKs.

## Conclusions

In conclusion, this study on acquired palmoplantar keratodermas (PPKs) represents a significant contribution to the field of dermatology by providing comprehensive insights into the diagnosis and management of these dermatological conditions. The study has established reliable diagnostic criteria for distinguishing between various types of acquired PPKs through meticulous analysis of clinical, dermoscopic, and histopathological features. Furthermore, correlations between these features have been elucidated, offering valuable insights into the underlying pathophysiology and guiding clinical decision-making. Additionally, the study has demonstrated the effectiveness of standard therapies, particularly for palmoplantar psoriasis, in improving disease severity and symptomatology. By providing evidence-based findings, this study informs the development of guidelines and protocols that enhance patient care and improve the quality of life for individuals affected by acquired PPKs. The knowledge gained from this study will continue to shape clinical practice, leading to more accurate diagnoses, tailored treatment approaches, and better outcomes for patients with acquired PPKs.
